# Urban 3D building morphology and energy consumption: empirical evidence from 53 cities in China

**DOI:** 10.1038/s41598-024-63698-1

**Published:** 2024-06-05

**Authors:** Yang Wang, Guiquan Sun, Yingmei Wu, Mark W. Rosenberg

**Affiliations:** 1 Faculty of Geography, Yunnan Normal University, Kunming, 650500 China; 2 Department of Geography, Queen’s University, Kingston, Ontario K7L 3N6 Canada

**Keywords:** 3D building morphology, Building energy consumption, Energy conservation, Electricity consumption, Spatial Durbin model, Large cities in China, Environmental social sciences, Energy and society, Sustainability

## Abstract

The impact of building morphology on building energy consumption has been extensively studied. However, research on how 3D building morphology affects energy consumption at a macroscopic scale is lacking. In this study, we measured the mean building height (BH), mean building volume (BV), and mean European nearest neighbor distance (MENN) of the city to quantify the 3D building morphology. We then used a spatial regression model to analyze the quantitative impact of urban 3D building morphology on per capita electricity consumption (PCEC). Results indicate that at the macroscopic scale of the city, the BH and the MENN have a significant positive impact on the PCEC, while the BV has a significant negative impact on the PCEC. Moreover, the inclusion of the 3D building morphology greatly improves the model’s ability to explain building energy efficiency, surpassing the impact of traditional economic factors. Considering the 3D building morphology indicators together, buildings with a lower height, a larger volume, and a more compact 3D morphology have greater potential for energy savings and are more conducive to electricity conservation. This study offers valuable insights for the energy-efficient arrangement of buildings.

## Introduction

The sustainability of cities is crucial for human survival and development because it impacts all aspects of human life. Cities consume over two-thirds of the world’s energy and generate a significant amount of pollutants and CO_2_^1^. Specifically, buildings contribute to 40% of the global energy consumption and account for 28% of the carbon emissions^2^. China’s “Total Energy Use Control” program aims to limit the national energy consumption to 6000 Mtce by 2030, encompassing all sectors, including the building sector. The objective of this plan is to effectively manage energy use in China, to reduce carbon emissions, and increase the proportion of non-fossil primary energy to 20% by 2030^3^.

Recent research by scholars in the field of buildings and energy has shifted focus from solely examining the buildings themselves to exploring energy conservation through the morphology of buildings. As global urbanization accelerates and population sizes continue to grow, the impact of building form on electricity consumption has become increasingly significant. Developed countries and regions have started to recognize the energy-saving potential of building form and have implemented various measures to enhance building energy efficiency and reduce power consumption. For instance, several European countries have enforced stringent building energy efficiency standards and green building policies, leading to the optimization of building forms and the adoption of energy-saving technologies, ultimately resulting in a reduction in electricity consumption. China, as one of the world's largest energy consumers, places significant importance on the construction industry in terms of energy consumption. The rapid urbanization process in China has led to a rise in the number of buildings, consequently increasing energy consumption during property development and utilisation of buildings. Traditional building designs often result in energy waste and high power consumption, highlighting the necessity of optimizing building design and planning to enhance energy efficiency. Moreover, China's energy structure is not environmentally friendly, with electricity generation, transportation, and usage impacting the environment. The potential energy efficiency of building forms directly influences environmental quality and sustainable development, underscoring the importance of promoting building energy efficiency and green building practices to mitigate environmental impact. Furthermore, the Chinese government's goal of establishing a resource-saving and environmentally friendly society can be supported by scientific research like this, providing a basis for formulating and implementing policies that promote building energy efficiency and sustainable development.

## Literature review

In recent years, significant focus has been given to the relationship between buildings and energy in the context of sustainable urban development. Understanding the relationship between buildings and energy along with its development mechanisms is crucial^4^. The energy consumption of buildings can be categorized into two main aspects: pre-manufacturing (during construction) and post-manufacturing (when the building is put into use)^5^. The energy consumption for manufacturing buildings is mainly consumed by the production of building materials and the building construction industry^6^. For example, Yang et al.^7^ constructed a model to save energy in the manufacturing of construction materials through a stereolithography process. Their experiments validated the effectiveness of the model in maintaining product quality while significantly reducing energy consumption. In the construction industry, Zhang et al.^8,9^ developed a process-based life cycle assessment model and found that the energy consumed in China’s construction sector from 2000 to 2016 quadrupling, representing approximately 9% of the total social energy consumption. They concluded that excessive and repetitive construction practices lead to unnecessary energy waste. In addition, building design can affect residential electricity consumption. In terms of energy conservation, the term “zero energy buildings” refers to energy-efficient buildings. Although this concept has met skepticism since its proposal, a new standard for zero-energy buildings has been proposed^10^. Several scholars have contributed to the theory of zero-energy buildings. Zhai et al.^11^ introduced a multi-objective optimization score method to optimize building window parameters, thereby improving the thermal environment and realizing energy conservation. Bui et al.^12^ utilized the firefly algorithm to predict the heating and cooling energy consumption of buildings, provided valuable guidance for designing energy-efficient buildings.

Residential living is a major energy consumer. Factors such as residential electricity consumption behavior and habits^13,14^, energy prices^15,16^, and appliance power consumption^17^ have an impact on energy consumption. However, residential electricity consumption behavior can only be studied on a small scale through simulation experiments and energy consumption model construction. For example, Pisello et al.^18^ conducted a study on the influence of personal attitudes on the energy demand of office buildings, using a university office building as a case study. Their findings reveal the significant impact of occupants’ behavior on building energy utilization. Similarly, Zhao et al.^19^ examined the interaction between energy-efficient technologies and occupants’ behavior using 300 residential buildings. Their study confirms the feasibility of energy savings from resident behavior, highlighting the potential for energy savings through technological advancements and residents’ behavior. Additionally, Fitzpatrick et al.^20^ investigated the benefits of real-time pricing in reducing electricity costs and enhancing energy supply flexibility, focusing on a residential building as their research subject. Given the limited scale of energy consumption detection within buildings, the methods employed can be enhanced through networked intelligent monitoring and mathematical modeling. However, the energy metrics within buildings also have several limitations, including the inability to expand the scope of the study and the study population. Despite the challenges in measuring the residential electricity consumption behavior, the building environment affects the behavior of residents' consumption of electricity. By measuring the external environment surrounding the building, researchers can shift their perspective from inside to outside, thereby broadening their outlook and improving the convenience of the study.

The electricity consumption behavior of residents is indirectly influenced by the external building environment, which in turn affects energy consumption^21–23^. Wang et al.^24,25^ examined the optimal layout for building energy conservation by analyzed three-dimensional metrics related to buildings, land use, and roadways. Their findings suggest that incorporating water bodies into the environment can contribute to energy conservation. Skelhorn et al.^26^ studied building energy changes due to urban densification and vegetation in Manchester, UK. Their research demonstrated that the addition of 5% more trees resulted in a 1 °C reduction in the peak urban heat island effect and a 4.8% decrease in energy consumption after 3 days of the peak heat island effect^22,27^ conducted a study in Nanjing, China, where they analyzed the vegetation pattern of around 40 buildings. In their study, the authors proposed a synergistic simulation technique that combines urban climate and energy. They focused on constructed vegetation morphology and argued that vegetation morphology has an impact on urban energy. In addition to the natural environment, the physical design of urban buildings also has a significant impact on energy utilization. This suggests a relationship between the physical morphology of urban buildings, such as compact cities and 3D building structures, and their ability to conserve energy^28,29^.

In their study, Shareef and Altan^30^ focused on urban neighborhoods and found that the arrangement of meandering neighborhoods and buildings can effectively regulate the outdoor microclimate and decrease energy utilization. Similarly, Leng et al.^31^, conducted research in Harbin, a cold region in China, to examined the environmental mechanisms by which building morphology affects energy utilization. They analyzed seven physical morphology indicators, including building coverage, floor area ratio, building height, and shape factor. Their results indicated that a high floor area ratio led to a 6.76% reduction in heating energy utilization, while the increase in the average building height to road width ratio on both sides of the road resulted in a 12.76% decrease in heating energy consumption. Other scholars have also considered additional indicators to measure building morphology. Li et al.^21,23^ incorporated building density, sky view factor, and building shadows as building morphology indicators. Their found indicate that building density contributes to the heating effect in spring while building shadows have a cooling effect in winter. Consequently, the inclusion of various measures of building morphology can have different impacts on building energy.

The existing literature primarily examines energy-saving mechanisms related to the external natural environment of buildings and building layout. These investigations mainly involve localized microscopic simulation experiments and measurements. Researchers have examined a limited number of neighborhoods, buildings, and individual cities to explore the external building environment, but macro-scale studies encompassing multiple cities are lacking. In the field of energy conservation, most research on building morphology indicators has primarily focused on 2D spatial morphology. However, there is still limited exploration and construction of three-dimensional morphological indicators. The research is notable for its extensive scope, sample size, and consideration of various building morphology dimensions. To investigate the impact of the external environment on energy conservation in buildings, we obtained 3D building data from 53 municipal districts in China in 2016. Our research specifically investigates the factors that influence building energy conservation by applying spatial regression methods and analyzing the perspective of 3D building morphology. The marginal contribution of this paper is that it is possible to analyse how the 3D morphology of buildings reduces energy consumption in terms of socio-economic and natural environmental factors, and that this research has a macro-guidance, which is useful for the government in the development of regulations for urban planning.

## Research methods

### Calculation method of building morphology index

Drawing on relevant literature, we selected mean building height (BH), mean building volume (BV), and mean European nearest neighbor distance (MENN) as the 3D morphological indicators of urban buildings, which are calculated as follows^32,33^.

(1) Mean building height

1 $$TB = \frac{{\sum\limits_{j = 1}^n {{A_j}{F_j}} }}{{n_j}}$$

where* A* is the floor area of the building, *n* is the total number of buildings within the city, *F* is the number of floors, and *j* represents the buildings within the city.

(2) Mean building volume

2 $$BV = \frac{{\sum\limits_{j = 1}^n {3{A_j}{F_j}} }}{{n_j}}$$

where *A* is the floor area of the building. When we performed the regression analysis, the *BV* was not log-standardized, probably because the variation in *BV* between cities was small and unaffected by heteroskedasticity. All variables were standardized except for *BV*.

(3) Mean Euclidean nearest neighbor distance

3 $$MENN = \frac{{\sum\limits_{j = 1}^n {d_j} }}{{n_j}}$$

where *d* is the distance between adjacent buildings.

### Ordinary least squares

Ordinary least squares (OLS), a classical linear regression model, was used to analyze the linear relationship between the 3D building morphology and the PCEC. OLS assumes that the independent variables are not correlated with each other, meaning that no covariance exists. However, OLS does not consider the interactions between the PCEC in spatially neighboring cities. The OLS model is expressed as follows^34^:

4 $${y_i} = \beta {X_i} + {\varepsilon_i},{\kern 1pt} {\kern 1pt} {\kern 1pt} \left[ {{\varepsilon_i}\ N(0,{\delta^2}I)} \right]$$

where *i* denotes the sample size of Chinese cities, *y* is the explanatory variable of the model, *X* is the influence factor of the PCEC, *β* denotes the regression coefficient of the influence factor, *ε* is the random error term of the model, *I* is the unit matrix, and *ε*_*i*_ ~ *N*(0,*δ*^2^*I*) indicates that the error term must follow normal distribution.

### Spatial regression model

The spatial lag model (SLM) considers the influence of explanatory variables on themselves or other explanatory variables in the same region or neighboring regions. The SLM is expressed as follows^8,9,35^:

5 $${y_i} = \rho \sum\limits_{j = 1}^n {{W_{ij}}{y_j}} + \beta {X_i} + {\varepsilon_i},{\kern 1pt} {\kern 1pt} {\kern 1pt} \left[ {{\varepsilon_i}\ N(0,{\delta^2}I)} \right]$$

where *ρ* denotes the spatial autoregressive coefficient value, and *W*_*ij*_ represents the spatial weights.

The spatial error model (SEM) incorporates the spatial correlation of random disturbance terms. The SEM is expressed as follows:

6 $${y_i} = \lambda \sum\limits_{j = 1}^n {{W_{ij}}{\varphi_i} + \beta {X_i} + {\varepsilon_i}} ,{\kern 1pt} {\kern 1pt} {\kern 1pt} \left[ {{\varepsilon_i}\ N(0,{\delta^2}I)} \right]$$

where* φ* is the error term affecting the PCEC of urbanites, and *λ* is the spatial autocorrelation coefficient of the error term.

The spatial Durbin model (SDM) is a combined extended form of the SLM and the SEM, considering both the spatial relationship of the dependent variable and the spatial relationship of the independent variable, which can be interpreted in this paper as the PCEC of a city is not only influenced by the PCEC of the surrounding cities but also by the independent variable of the neighboring cities. The SDM is expressed as follows^36^:

7 $${y_i} = \rho \sum\limits_{j = 1}^n {{W_{ij}}{y_j}} + \lambda \sum\limits_{j = 1}^n {{W_{ij}}{\varphi_i}} + \beta {X_i} + {\varepsilon_i},{\kern 1pt} {\kern 1pt} {\kern 1pt} \left[ {{\varepsilon_i}\ N(0,{\delta^2}I)} \right]$$

where the various symbols have the same definitions as those in Eqs. (5) and (6).

### Ethical approval

This article does not contain any studies with human participants performed by any of the authors.

### Informed consent

This article does not contain any studies with human participants performed by any of the authors.

## Research data and framework

### Study area

**Figure 1:**
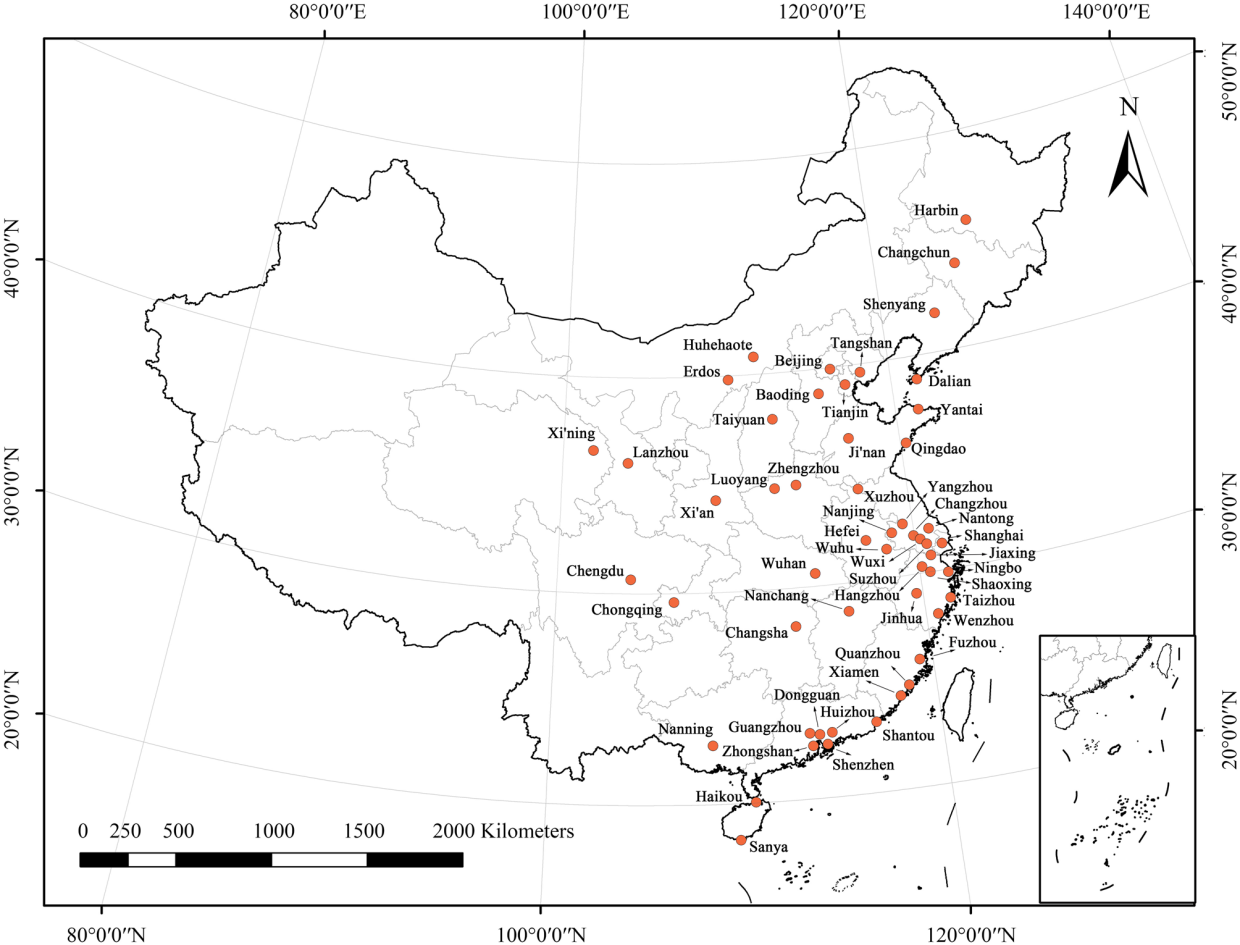
Study area overview.

The study region is summarized in Fig. 1. The study unit is the municipal districts of 53 major cities in China, which are the main geographic areas. Compared with other county-level administrative districts, municipal districts are the core component of city (i.e., the urban area) and the center of regional economic development. In municipal districts, there is typically a high degree of urbanization, resulting in high population density and a relatively concentrated mobile population. The study unit includes 8 eastern provincial capital centers, 5 central provincial capital centers, 4 western provincial capital centers, 4 municipalities directly under the central government, and the remaining 32 prefecture-level cities, and 50 of these areas are large cities (urban areas with a population greater than 1 million). The study area covers most of the provincial capital cities, which is representative of the macro-regional study scale perspective.

### Research path

**Figure 2:**
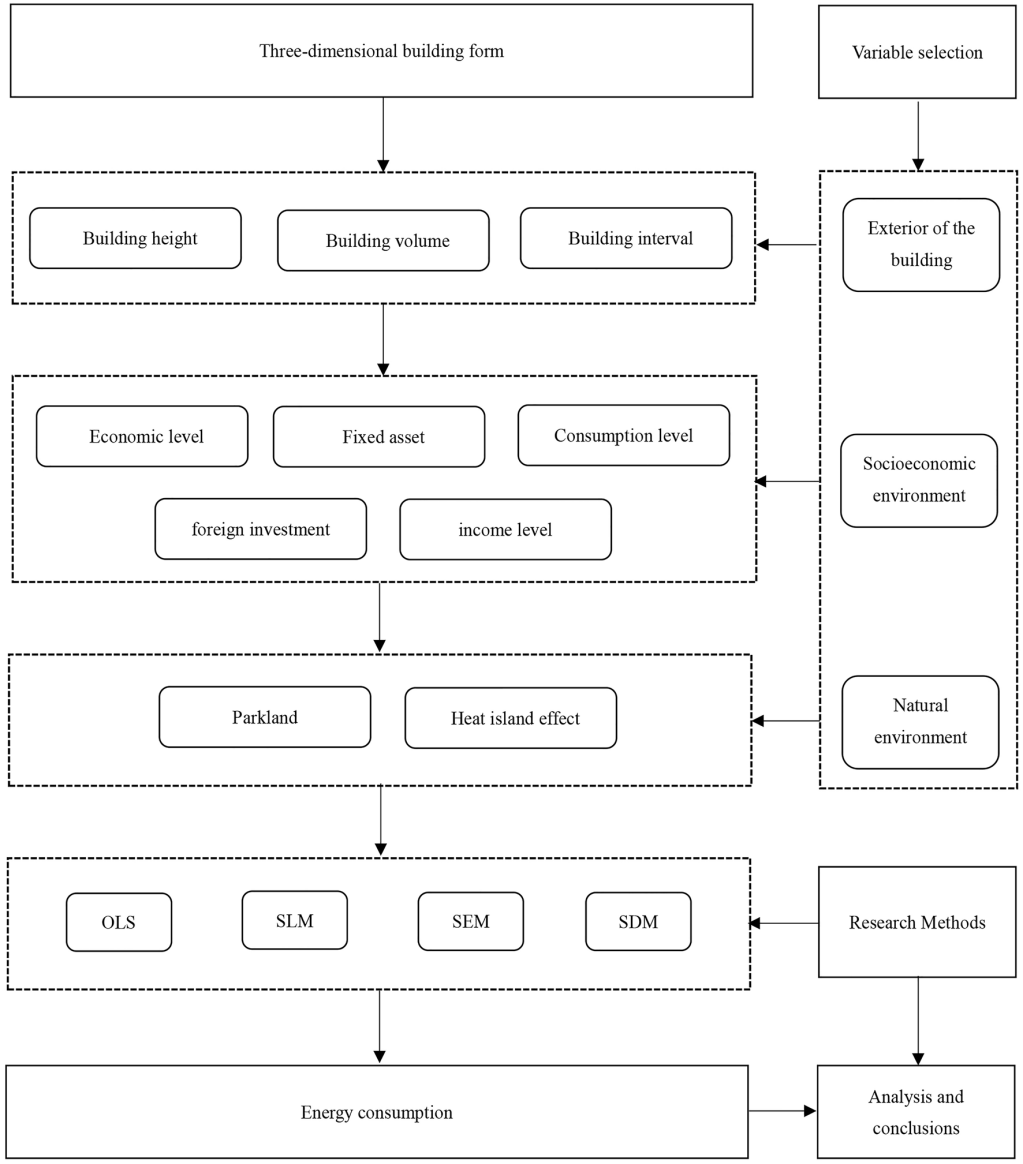
Research path.

After drawing on the theoretical frameworks and research methods of previous researchers, we constructed the research framework according to the actual situation of this study, Fig. 2 shows the technology roadmap of the research in this paper^27,37–39^. In this study, we constructed a comprehensive model of building form using 3D building form indicators. In addition, we considered socioeconomic factors and natural environment factors. We then conducted regression analyses to determine the effects of 3D building form and socioeconomic variables on per capita electricity consumption (PCEC).

### Data sources

The 3D building vector data were obtained from Baidu Map (2016), and we estimated the height of each floor as 3 m. The electricity data for this article comes from the 2017 China Energy Statistics Yearbook. Indicators related to socioeconomic factor variables (control variables) were obtained from the 2017 China Statistical Yearbook and the 2017 China City Statistical Yearbook (2016 data).

### Variable selection

#### Dependent variable

The electricity consumption of the entire society encompasses the combined electricity consumption of each industry and that of urban and rural residents. As this paper focuses on 3D buildings, which encompass all building types, including those specific to each industry, the PCEC was utilized as the explanatory variable.

#### Explanatory variables

In this study, we utilized BH, BV, and MENN as the three representative indicators to describe the 3D morphology of buildings^15,16,40^. The variable BH denotes the mean height of urban buildings, while BV and MENN represent the compactness of the urban building morphology.

#### Control variables

The social factors affecting PCEC are diverse, and six control variables were selected based on the relevant literature. Per capita GDP can represent the standard of living of the population to some extent, and the standard of living has an important impact on energy consumption^41^. Fixed asset investment can reflect the industrial structure of a region, and for developing China, large machines and projects are among the main consumers of energy^42^. The use of foreign capital can measure the degree of openness of a region to a certain extent, and we use the per capita amount of actual foreign capital utilized to express the degree of openness of the region; furthermore, studies have shown that a causal relationship exists between capital flows and energy consumption^43^. The average wage of employees represents the per capita income level of residents, and the income level affects the residents’ electricity consumption habits, thus indirectly affecting the household electricity consumption^44^. Based on the literature that urban landscapes, such as vegetation and water bodies, play an important role in regulating the microclimate of a city, we used per capita green space in parks to represent a control variable that affects building energy consumption.

## Results and discussion

### Differences in 3D building morphology and per capita electricity consumption fluctuations in 53 major cities in China

**Figure 3:**
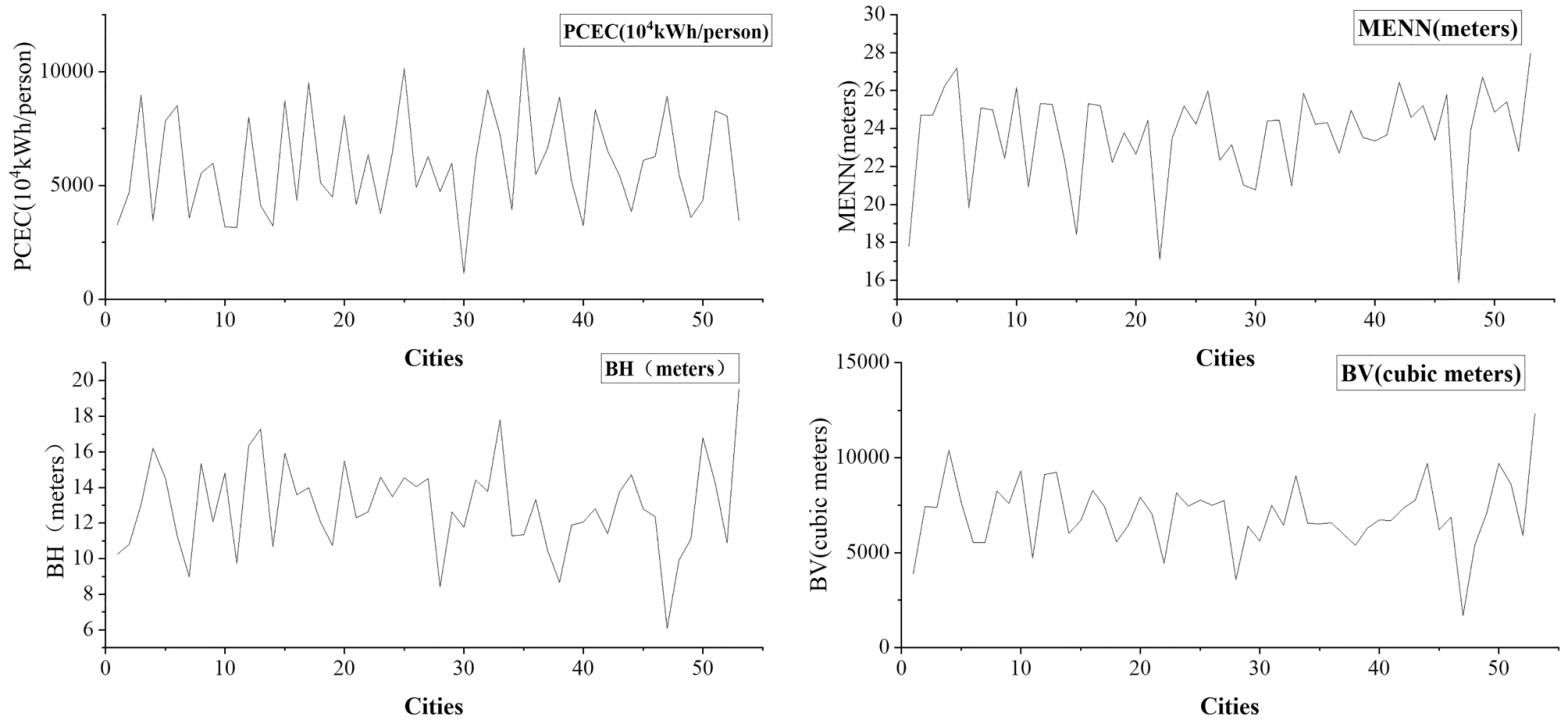
Differences in PCEC and building morphology fluctuations.

We used line graphs to differentiate the building morphology indicators and the PCEC for 53 cities. In Fig. 3, the PCEC fluctuates remarkably between each city. The fluctuations in the BV are also large, with small fluctuations in BV overall. Three high peaks in MENN can be observed, except for the small fluctuations in MENN between cities.

**Figure 4:**
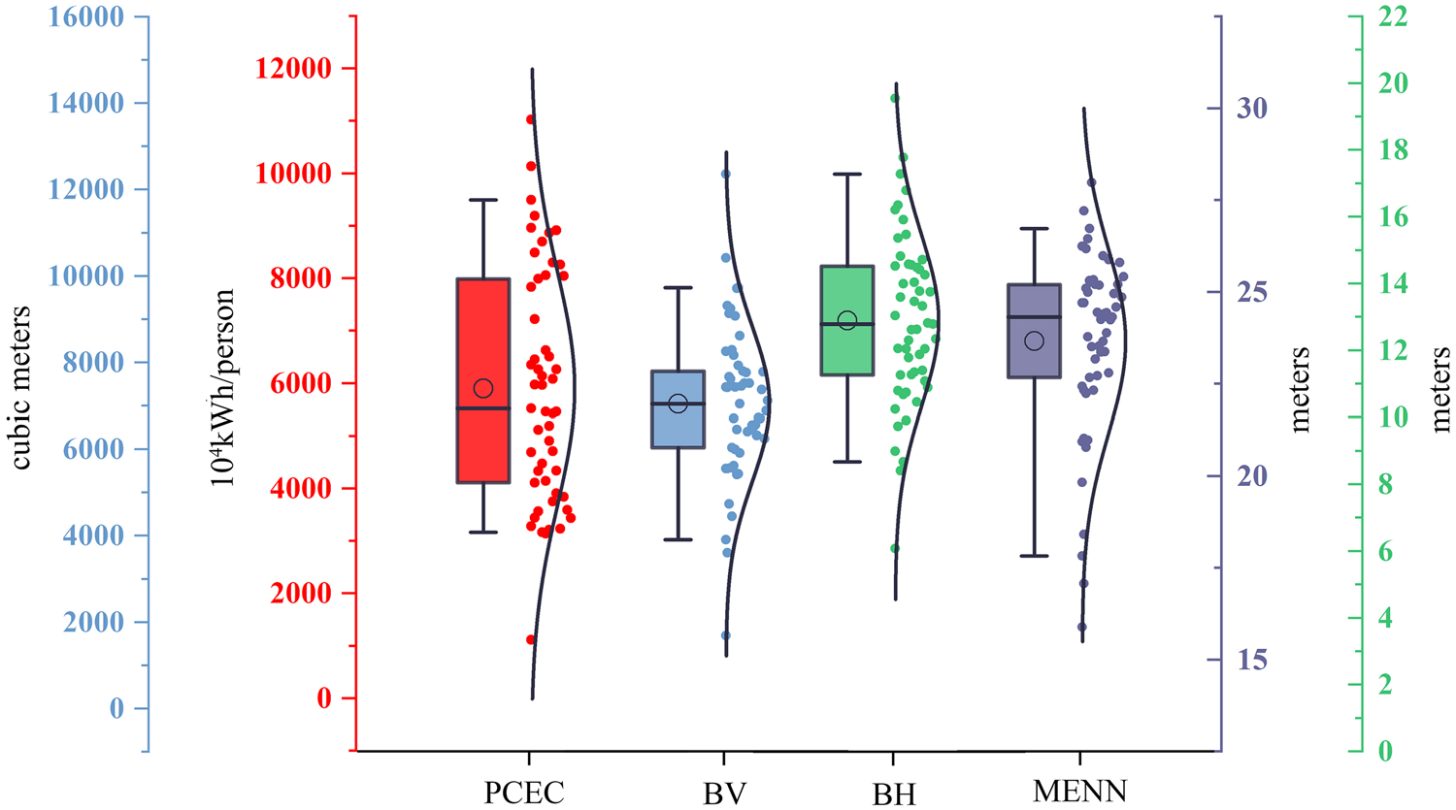
Description of PCEC and building morphology data.

We present box plots of the overall differences between the PCEC and the building morphology metrics, with the data for each metric consisting of two elements, left and right. On the left, the whisker line range represents the 5% to 95% percentile (Fig. 4). The circles in the box represent the mean, and the horizontal line represents the median. On the right, the curves and the dots represent the normal distribution of the data.

**Figure 5:**
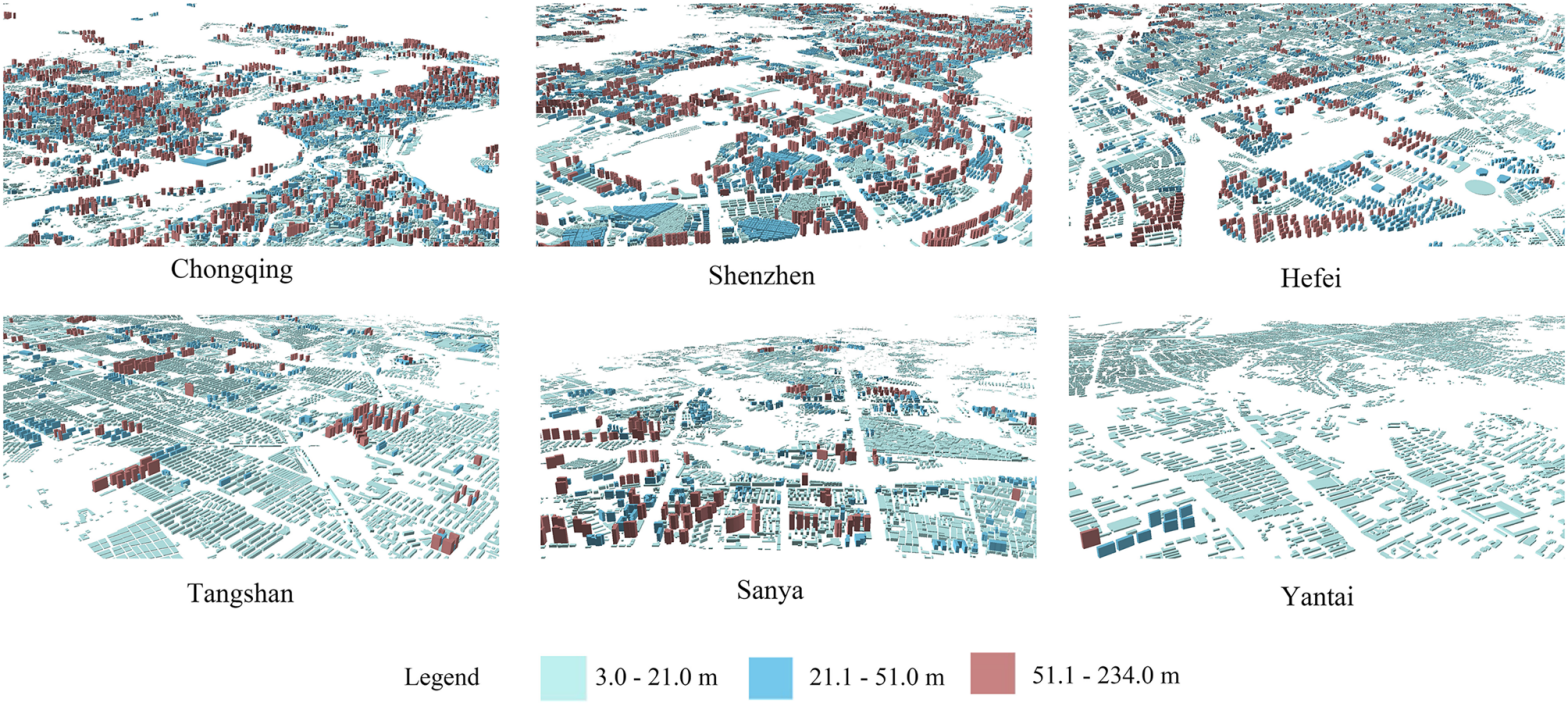
Typical city 3D building local display chart.

We show the 3D maps of the buildings in each of the six cities in Fig. 5 and have classified the heights of the buildings into three classes, with the first class ranging from 3 to 21 m, the second class ranging from 21 to 51 m, and the third class ranging from 51 to 234 m. In Fig. 5, darker building colors represent higher building heights, and conversely, lighter building colors represent lower buildings.

**Figure 6:**
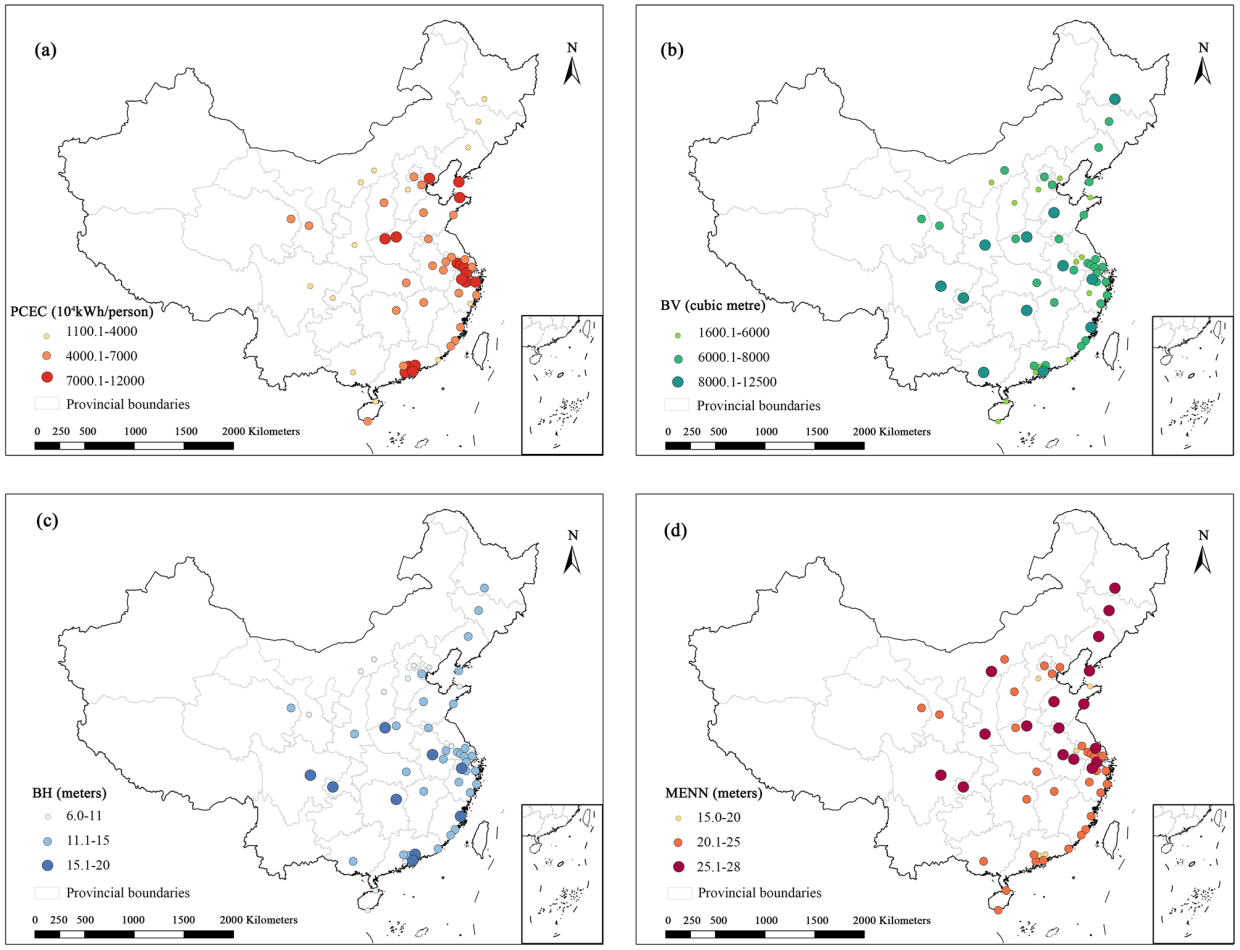
Spatial variation in PCEC, BV, BH and MENN.

To compare building heights and distances across cities, we utilized ArcGIS software to visually represent PCEC, BH, BV, and MENN. Figure 6 illustrates significant differences in these aspects among the Yangtze River Delta (Shanghai), the Bohai Economic Circle (Beijing), and the Pearl River Delta (Guangzhou), highlighting clear spatial variations compared to other cities. In Figures (b) and (a), The BH and BV of the three major economic regions generally fall within the medium range compared to other cities. Contrary to expectations based on empirical perception, the average height of urban buildings in more developed regions tends to be lower. For example, Beijing's urban planning in 1993, as outlined in the Beijing Urban Master Plan (1991–2010), emphasized the protection of historic city areas. The plan specified controlled building heights in a hierarchical manner, with the Forbidden City and Imperial City as focal points. The old city was to maintain a spacious layout, with building heights gradually increasing from the center to the periphery, capped at 9 m, 12 m, and 18 m, respectively. However, many buildings still exceed these limits in practice. The MENN is lower in most coastal cities compared to other urban areas, possibly due to the concentrated land development in economically advanced cities. This, along with real estate developers maximizing profits by increasing plot ratios, results in tighter building spacing and a trend towards high-rise construction. In 2020, Shanghai introduced a policy to strictly control the expansion of construction land, emphasizing the reduction of construction areas without encroaching on ecological land.

### Impact of building morphology on PCEC

#### Impact of building morphology on PCEC: Based on OLS

We provide a short description of each variable (Table 1). We used Stata16 for the regression analysis. The BV was not log-standardized, probably because the variation in BV between cities was small and unaffected by heteroskedasticity. Thus, all the variables were standardized except for the BV.

**Table 1:** Descriptive statistics for each variable.

Variables	Definition	Unit	Min	Max
PCEC	Per capita electricity consumption	10^4^ kWh/person	7.024	9.306
BH	Mean building height	meters	6.097	19.55
BV	Mean building volume	cubic meters	1680	12,348
MENN	Mean European Nearest Neighbor Distance	meters	15.88	27.97
PCGDP	Per capita GDP	yuan/person	10.53	12.08
PCFAI	Per capita fixed asset investment	yuan/person	9.845	11.88
PCCG	Per capita retail sales of consumer goods	yuan/person	1.686	11.38
PCFCU	Per capita amount of actual foreign capital utilized	yuan/person	3.821	9.744
AW	Average wage	yuan/person	10.93	11.72
PCGSP	Per capita green space in parks	square metre/person	2.058	3.552

The BH has a significant positive effect on the PCEC. The MENN also has a positive effect on PCEC, indicating that the greater the distance between buildings is, the smaller the PCEC is (Table 2). In addition, the BV has a negative effect on the PCEC. Among the economic indicators, the per capita GDP has a significant positive effect on the PCEC, which is inextricably related to the massive expansion of infrastructure construction in the context of China’s rapid economic development in recent years.

**Table 2:** OLS results.

Variables	Coefficient	Standard Deviation	t value	*p* value
BH	0.088*	0.049	1.80	0.079
BV	− 0.0002**	0.000	− 2.21	0.033
MENN	0.034	0.034	1.00	0.322
PCGDP	0.862***	0.237	3.64	0.001
PCFAI	− 0.189	0.143	− 1.32	0.198
PCCG	0.027	0.044	0.62	0.539
PCFCU	0.047	0.054	0.88	0.385
AW	− 0.548	0.361	− 1.52	0.136
PCGSP	− 0.373	0.22	− 1.69	0.10
Constants	6.668	4.00	1.67	0.103

***p < 0.01, **p < 0.05, *p < 0.1 (Same below).

#### Impact of building morphology on per capita electricity consumption: based on SDM

Most regression models typically focus on causal relationships between variables, often overlooking the impact of spatial distance. In contrast, SDM incorporates the influence of distance on the analysis outcomes. In Table 3, the R^2^ of the SDM is higher than those of OLS, the SEM, and the SLM, and the log likelihood value of the SDM is the largest. Thus, the SDM was used for the result analysis, and OLS, the SLM, and the SEM were used as references for comparison analysis.

**Table 3:** Model Selection.

Models	OLS	SLM	SEM	SDM
R^2^	0.434	0.434	0.434	0.590
Log Likelihood		− 13.748	− 14.748	− 4.627

The analysis shows that for every 1% increase in BH, a 7.6% increase in the PCEC follows (Table 4). For every 1 m^3^ increase in BV, the PCEC decreases by − 0.02%, indicating that as the volume of the building increases, the PCEC decreases. Therefore, the lower the building height and the larger the volume is, the lower the energy consumption is. MENN represents the degree of compactness of a 3D building. A 1% increase in MENN increases the PCEC by 2.3%, indicating that the greater the mean distance between buildings is, the greater the energy consumption is; conversely, the smaller the mean distance between buildings is, the lower the energy consumption is. On the contrary, the smaller the mean distance between buildings is, the lower the energy consumption will be. In addition, the mean building height consumes more energy and the mean building volume consumes less energy than would otherwise be the case under the influence of spatial effects.

**Table 4:** SDM result.

Variables	Coefficient	Standard Deviation	t value	*p* value
BH	0.076*	0.428	1.77	0.076
BV	− 0.0002*	0.000	− 1.95	0.052
MENN	0.023*	0.033	0.71	0.476
PCGDP	0.940***	0.255	3.69	0.000
PCFAI	− 0.303**	0.133	− 2.27	0.023
PCCG	0.030	0.037	0.82	0.552
PCFCU	0.056	0.051	1.11	0.330
AW	− 1.048***	0.347	− 3.02	0.003
PCGSP	− 0.393*	0.205	− 1.92	0.055
Constants	12.715	3.675	3.46	0.001

We included each of the 3D building indicators in the model and used the economic factors as the control group. In Table 5, the traditional economic variables BH, BV, and MENN are added to Models 2, 3, and 4, respectively. The inclusion of 3D building morphology indicators (Models 2, 3, and 4) results in a larger R^2^ value than that in Model 1, indicating the increased explanatory power of the model. When the 3D building morphology indicators (BH, BV, and MENN) were added to Model 4, the significance of the coefficients of the 3D building morphology increased. Additionally, the Wald, F, and log likelihood values were larger in Model 1 than in the other four models (Models 1, 2, and 3). These findings further support the notion that the explanatory power of 3D building morphology for the SDM increased, highlighting its importance as a key influencing factor for energy conservation.

**Table 5:** Model comparison of BV, BH, and MENN with traditional economic variables.

Variables	Model1	Model2	Model3	Model4
BH		− 0.011	0.054	0.076*
		(− 0.59)	(1.63)	(1.77)
BV			− 0.0001**	− 0.0002*
			(− 2.26)	(− 1.95)
MENN				0.023
				(0.71)
PCGDP	0.971***	0.971***	0.960***	0.940***
	(3.77)	(3.73)	(3.77)	(3.69)
PCFAI	− 0.246*	− 0.260*	− 0.269**	− 0.303**
	(− 1.88)	(− 1.94)	(− 2.02)	(− 2.27)
PCCG	0.016	0.020	0.018	0.030
	(0.43)	(0.52)	(0.48)	(0.82)
PCFCU	0.019	0.024	0.055	0.056
	(0.36)	(0.44)	(1.06)	(1.11)
AW	− 1.039***	− 1.032***	− 0.982***	− 1.048***
	(− 3.08)	(− 3.07)	(− 2.99)	(− 3.02)
PCGSP	− 0.375*	− 0.407**	− 0.416**	− 0.393*
	(− 1.90)	(− 1.99)	(− 2.08)	(− 1.92)
Constant	12.466***	12.678***	12.058***	12.715***
	(3.62)	(3.65)	(3.64)	(3.46)
R2	0.450	0.454	0.566	0.590
Wald Test	32.711	31.556	46.965	49.008
F Test	2.726	2.254	2.935	2.732
Log Likelihood	− 10.452	− 10.274	− 5.805	− 4.627
Observations	53	53	53	53

### Discussion

In this study, four analytical models (OLS, SEM, SLM, and SDM) were constructed to analyze the relationship between building physical morphology factors and socio-economic factors. The results indicate that the SDM is the most appropriate model for theoretical expectations and demonstrates a statistical causal relationship between building morphology and PCEC. First, the higher the BH is, the higher the PCEC is due to the heat island effect, which leads to longer microclimate influence and sunlight hours in summer, which in turn increases the cooling demand. Similarly, taller buildings have a lower ambient temperature around them, leading to increased heating demand in winter. Therefore, the BH has a positive effect on the PCEC, and the findings of Xi et al.^45^ provide research support for the interpretation of this conclusion. Second, the larger the BV is, the lower the PCEC is. Therefore, when examining the relationship between the building volume and the PCEC, focus should be directed on increasing the flat area of the building and controlling the mean height because the BH has a negative effect on the PCEC. In theory, a large building volume improves ventilation in hot environments and reduces the heating demand. Third, in terms of building spacing, the smaller the separation between buildings is, the greater the energy efficiency is because a small spacing indicates a highly compact building morphology. A compact building group with a small spacing creates a large shadow area, which helps prolong the time of indoor warming and reduce the cooling demand in a solar thermal radiation environment. Similarly, in cold environments, a small building spacing is beneficial for preserving solar radiation and reducing the heating demand. These findings are consistent with those of the research conducted by Xie et al.^46^.

At the socio-economic level, per capita GDP represents the standard of living and prosperity of a region. The level of economic development influences the energy consumption patterns necessary for the population’s livelihood. This influence is pervasive and impacts various aspects of the population’s life, which is consistent with the findings of Duan et al.^47^. The impact of average wages on PCEC was found to be significantly negative. This outcome suggests that the increase in wages leads to an overall improvement in the standard of living for the population, resulting in increased purchasing power for energy-efficient appliances.

The per capita fixed asset investment has a negative impact on PCEC. In recent years, investment in energy-efficient fixed assets has gradually increased, which can indirectly improve energy efficiency and reduce energy consumption. Wang et al.^42^ examined the correlation between fixed assets and energy consumption in three major industries in China. They found that investment in fixed assets in the secondary industry increased energy consumption, while investment in fixed assets in the primary industry decreased energy consumption. The study also explains the negative impact of per capita fixed asset investment on PCEC by considering the current utilisation of per capita fixed asset investment in these industries. The government should consider increasing investment in energy-saving fixed assets within the secondary industry. Additionally, they can create policies and regulations to incentivize both enterprises and individuals to invest in energy-saving fixed assets. This can include implementing tax incentives and subsidy policies to lower investment costs and improve the return on investment. The per capita amount of actual foreign capital utilized has a positive effect on PCEC. According to Omri and Kahouli^48^, the increase in foreign investment promotes economic growth, which in turn attracts more foreign investment inflows. This increase in foreign investment eventually leads to the expansion of the industry, resulting in higher energy consumption, which aligns with the findings of this study. Furthermore, the per capita retail sales of consumer goods have a positive impact on PCEC. These sales reflect the living standards and consumption potential of the residents, enabling them to acquire electronic products and bulk goods, thereby indirectly contributing to the increase in energy consumption. Parkland has a negative impact on building energy consumption. Specifically, parkland reduces heat radiation from the urban heat island effect, thereby reducing the need for cooling energy in buildings. In the realm of urban planning, it is recommended that the Government enhance land development intensity to address urban development challenges and improve urban spatial quality. This involves trading intensity for space, elevating quality through space, and prioritizing the use of land freed up from intensified development for the creation of additional public green space, public areas, and service facilities. This approach aims to cultivate a more spacious urban spatial layout with lower population density.

This study has some shortcomings. First, the building morphological indicators and socio-economic factors discussed in this article are limited because various factors affect the building morphology and socio-economic factors of PCEC, making their quantitative measurement difficult. In future studies, including additional indicators, such as floor area ratio and lighting, may be beneficial to the analysis of building morphology. Second, this paper focuses on a sample of 53 large cities in China, excluding 661 cities. Additionally, the use of cross-sectional data for 2016 limits the ability to analyze future trends. Future research could consider using panel data to explore the energy-saving mechanisms of 3D building morphology. Finally, Our study does not rise to the level of energy-saving mechanisms, one major challenge is the difficulty in accurately measuring the complete building morphology. Our current findings suggest that during urban planning, relevant authorities should consider 3D building morphology factors, such as distance, height, and volume, between buildings or building groups. This perspective on energy efficiency can help in constructing an energy-efficient urban building morphology and increase the potential for energy savings through human intervention in the external building environment.

## Conclusions and policy implications

### Conclusion

This study aimed to examine the impact of building morphology on PCEC using cross-sectional data from 53 municipalities in China in 2016. Additionally, five socio-economic factors were considered as control variables to construct a spatial regression model. The regression model incorporated the scale effect from spatial distance. The results of the spatial regression analysis revealed that out of the 3D building morphology indicators, the BV was found to have a negative effect on the PCEC. Furthermore, a small MENN value was associated with a low PCEC, while a high BH was linked to a high PCEC. The analysis results of the 3D building morphology indicators indicate that buildings with a lower height, a larger volume, and a more compact morphology have a greater potential for energy saving and are more beneficial for energy conservation. Moreover, the inclusion of the 3D building morphology greatly improved the model’s ability to explain building energy efficiency, surpassing the impact of traditional economic factors.

### Policy recommendations

This study has policy implications. First, building complexes are impacted by the heat island effect. The demand for residential cooling and heating, elevator usage, and transportation increases with the BH, leading to increased energy consumption. Hence, the height drop of buildings should be considered when planning and designing urban buildings, rather than solely focusing on upward 3D development. This approach can help in reducing the mean height of buildings. In addition, in the context of designing buildings and urban planning, controlling the BH and increasing the building plan area should be considered. This approach can effectively increase the building volume and subsequently reduce the PCEC. Finally, the design of compact building morphology aims to minimize the average distance between buildings and prevent buildings from being scattered. This approach ensures proper ventilation and lighting while promoting energy conservation in cities. These three aspects offer valuable insights for designing energy-efficient building groups.

### Supplementary Information

Supplementary Information.

## Data Availability

Data is provided within supplementary information files.
